# Corneal plates of fluoroquinolone

**DOI:** 10.11604/pamj.2014.18.249.4790

**Published:** 2014-07-26

**Authors:** Mohammed El Mellaoui, Abdelkader Laktaoui

**Affiliations:** 1 Ophthalmological Service, Moulay Ismaïl Military Hospital, Meknes, Morocco

**Keywords:** Corneal plates, corneal deposits, fluoroquinolone

## Image in medicine

A 55-year-old man operated on for tractional retinal detachment secondary to proliferative diabetic retinopathy, receives topical treatment including ciprofloxacin eye-drops and ointment. Lost sight of for a month without stopping treatment, patient returns with a real crystallization of the cornea as central whitish and hard plates corresponding to deposits of fluoroquinolone. The treatment was stopped immediately and intense lubrication has been prescribed for two days to facilitate debridement of residual débrits. The évolution was marked by the persistence of a corneal opacification with impact on final visual acuity. The abusive use of fluoroquinolones must be avoided and it is wished that drugs for ophthalmic use are going to be less toxic to eye.

**Figure 1:**
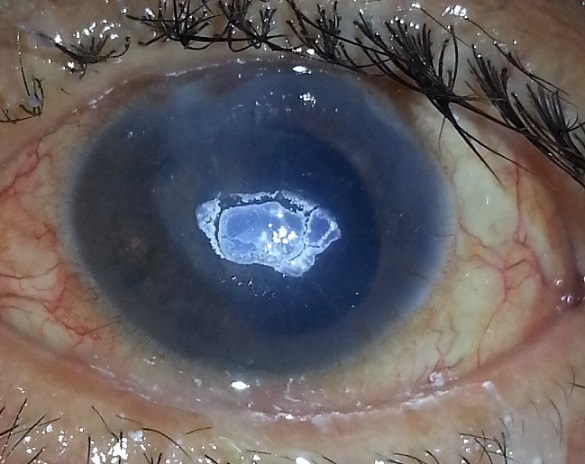
Corneal deposits of fluoroquinolone

